# Comparison of coronal and sagittal alignment in patients without osteoarthritis but with knee complaints

**DOI:** 10.1002/jeo2.70165

**Published:** 2025-02-10

**Authors:** Mikiko Handa, Tsuneari Takahashi, Akihiro Saitsu, Masaki Iguchi, Katsushi Takeshita

**Affiliations:** ^1^ Department of Orthopedics Jichi Medical University Shimotsuke Japan; ^2^ R & D Division of Career Education for Medical Professionals, Medical Education Center Jichi Medical University Shimotsuke Japan; ^3^ Department of Orthopedic Surgery Miyazaki Prefectural Nobeoka Hospital Nobeoka Japan

**Keywords:** coronal alignment, CPAK classification, knee, knee osteoarthritis, lower limb alignment, sagittal alignment

## Abstract

**Purpose:**

This study aimed to clarify lower limb alignment characteristics and the relationship between coronal and sagittal lower limb alignment in Japanese patients with knee complaints but without knee osteoarthritis (KOA).

**Methods:**

This retrospective study included 200 knees from Japanese patients with knee complaints but without KOA aged 60 years and under presenting with knee complaints between May 2020 and May 2023. We assessed coronal parameters, including mechanical hip knee angle (mHKA), arithmetic HKA (aHKA) mechanical lateral distal femoral angle and medial proximal tibial angle (MPTA), and sagittal parameters, such as medial posterior tibial slope (MPTS) and lateral posterior tibial slope (LPTS). In addition, we compared differences between CPAK types.

**Results:**

A weak positive correlation was found between mHKA and MPTS, but not with aHKA. Moderate negative and moderate positive correlations were found between mHKA and MPTA and between medial and LPTS, respectively.

**Conclusions:**

Little correlation was found between the coronal and sagittal parameters in patients with knee complaints but without KOA.

**Level of Evidence:**

Level Ⅲ.

**Abbreviations**
aHKA: arithmetic hip knee angle
CPAK: coronal plane alignment of knee
ICC: intraclass correlation coefficient
KA: kinematic alignment
KOA: knee osteoarthritis
LPTS: lateral posterior tibial slope
MA: mechanical alignment
mHKA: mechanical hip knee angle
mLDFA: mechanical lateral distal femoral alignment
MPTA: medial proximal tibial alignment
MPTS: medial posterior tibial slope
PTS: posterior tibial slope
TKA: total knee arthroplasty

## INTRODUCTION

The mechanical alignment (MA)‐total knee arthroplasty (TKA) alignment differs from the natural anatomy because the natural joint line is inclined at a mean of about 3°. Generally, the native alignment of the lower limb is not a neutral alignment. Particularly, Asians tend to have a larger tibial varus and a higher knee osteoarthritis (KOA) prevalence [8, 9]. Wanezaki et al. reported that healthy Japanese lower limb alignment tended to have a smaller mechanical lateral distal femoral angle (mLDFA), medial proximal tibial angle (MPTA) and larger mechanical hip knee angle (mHKA) compared with other countries. The rates of constitutional varus (mHKA varus ≥3°) tended to be higher than those in other countries [22]. The effectiveness of correcting the native alignment, which is a neutral alignment for MA alignment, remains unclear [12].

In addition to coronal alignment, sagittal alignment should be considered. As the posterior tibial slope (PTS) increases, the posterior tibial subluxation increases and the quadriceps muscle strength is reduced [18]. PTS is approximately 10° medially and 8° laterally; due to individual differences, it differs from the TKA implant makers' recommended PTS [17, 18]. In recent years, restoring to the patient's native alignment has also gained support, and kinematic alignment (KA)‐TKA has increased [14]. The advantages of KA‐TKA are restoring the natural joint line and PTS, and soft tissue release is lesser than with MA‐TKA. However, its effectiveness remains uncertain, as some reports indicate that pronounced tibial varus might influence long‐term outcomes [1, 7, 9].

Most studies of lower limb alignment have focused on assessing coronal alignment in healthy volunteer groups, with few studies assessing sagittal alignment. To the best of our knowledge, no study has compared coronal lower limb alignment with sagittal lower limb alignment in patients with knee complaints but without KOA. The purpose of this study was to clarify the hypothesis that there is no correlation between coronal and sagittal alignment in patients with knee complaints but without KOA.

## METHODS

The institutional review board approved this study. This retrospective study included Japanese males and females aged ≤60 years who visited our outpatient clinic for knee‐related complaints between May 2020 and May 2023. Age, sex, height, weight and medical history were determined, and full leg‐standing radiographs and lateral radiographs were obtained. All patients agreed to undergo radiography. The exclusion criteria were patients who had osteophyte or joint space narrowing, a history of knee surgery, young patients with bone immaturity (Risser sign ≤ 3) and failure to obtain adequate anterior–posterior and lateral views on radiographic assessment. We examined 200 knees: 105 males (mean age, 35.7 ± 14.0) and 95 females knees (mean age, 38.3 ± 16.0).

For the primary endpoint, we measured the radiographs to correlate the coronal and sagittal parameters (coronal parameters: mHKA, mLDFA, MPTA; sagittal parameters: medial posterior tibial slope [MPTS], lateral PTS [LPTS]). In addition, we used each value's mean and SE to calculate 95% confidence intervals (CIs) for the population and compared them with the values of the previous Japanese study. The Coronal Plane Alignment of Knee (CPAK) classification system was used to assess lower limb alignment characteristics by joint line obliquity and arithmetic HKA (aHKA) [6, 13].

In the subgroup analysis, patients with knee complaints were followed up and divided into three subgroups (noninjury, intra‐articular injury and extra‐articular injury groups) based on the presence and location of the injury. The parameters and CPAK classification were compared in each of the three groups.

### Measurements

Radiographs were obtained using the method of Paley et al. [16]. We set the distance between the cassette and tube to 300 cm, the tube voltage to 85 kV and the tube current to 200 mA. We obtained the radiographs in a standing position with the patella oriented forward and lateral. The radiograph length was 25.4 × 30.5 cm, and the lateral view of the tibia was taken showing the proximal 1/2 of the tibia.

**Figure 1:**
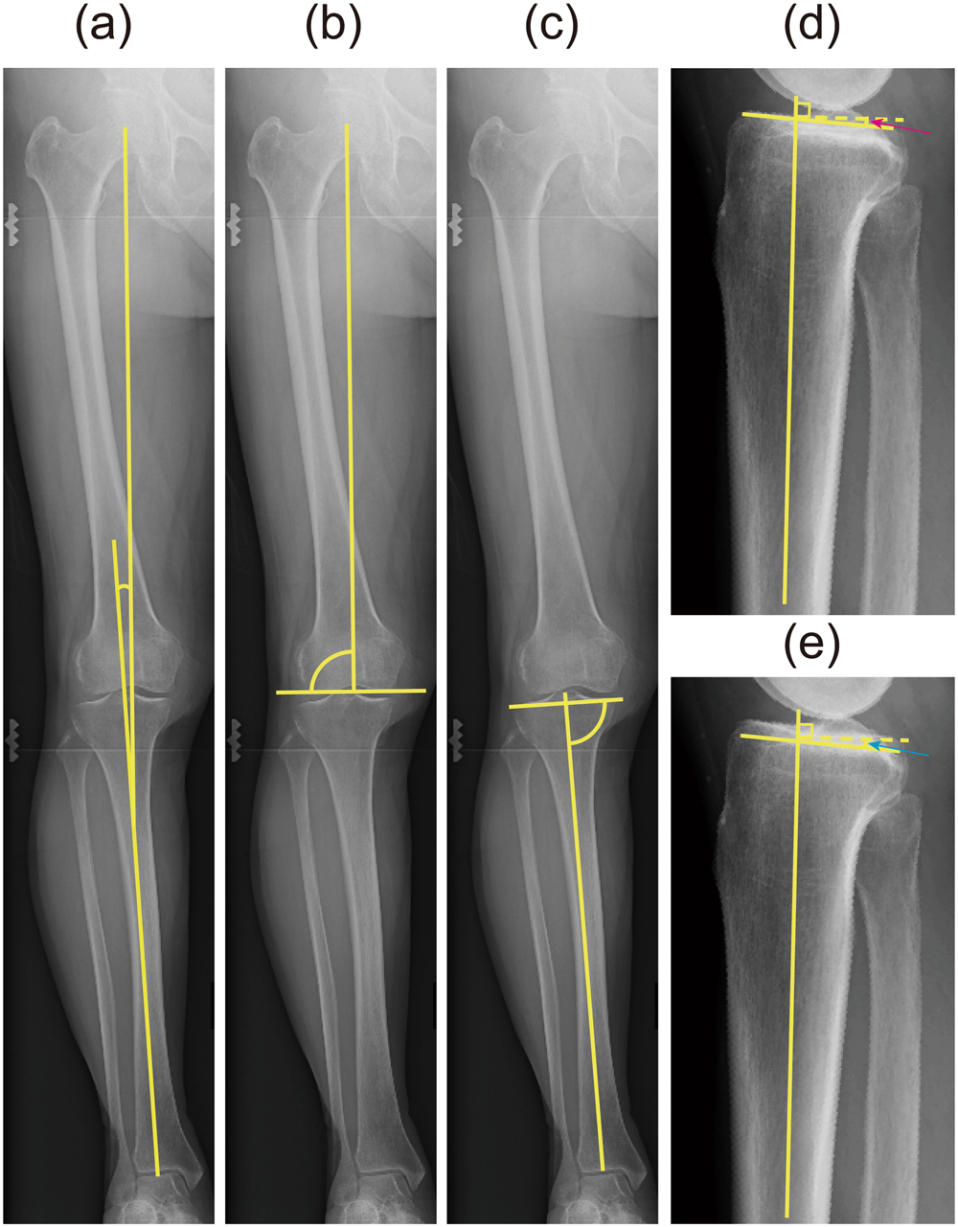
Measurement of the parameters. (a) Mechanical hip knee angle. (b) Mechanical lateral distal femoral angle. (c) Medial proximal tibial angle. (d) Medial posterior tibial slope: a pink arrow. (e) Lateral posterior tibial slope: a blue arrow.

We measured mHKA, mLDFA and MPTA using PACS (PSP Co.). mHKA was defined as the angle of the mechanical femoral and tibial axes, with positive and negative values for valgus and varus, respectively. aHKA was defined as MPTA‐mLDFA. mLDFA was defined as the lateral angle of the mechanical femoral axis and the femoral joint line. MPTA was defined as the medial angle of the tibial joint line and the mechanical tibial axis. In measuring the MPTS and LPTS from the lateral radiograph of the tibia, the medial and lateral tibial plateaus were defined as larger and smaller square protrusions of the posterior margin, respectively [19]. Regarding the anatomical tibial axis, the MPTS was the angle between the anatomical axis and the medial tibial plateau, and the LPTS was the angle between the anatomical axis and the lateral tibial plateau, the angle of difference between the anatomical axis and a line perpendicular to it. The measurement methods were performed based on previous studies [1, 3, 22]. mHKA with >3° of varus and >3° of valgus was defined as constitutional varus and constitutional valgus, respectively (Figure 1) [1, 20, 21]. We excluded those with osteophyte or joint gap narrowing, a history of knee joint surgery, young patients with a Risser sign 4 and no bone maturation, and with incorrect anterior–posterior view. We classified constitutional lower extremity alignment characteristics using the CPAK classification [6, 10].

### Statistical analysis

Data analysis was performed using G*Power and EZR. The Shapiro–Wilk test, Fisher's exact test, *t* test and meta‐analysis were performed for the primary endpoint. Statistical significance was defined as *p* < 0.05. Post hoc analysis was performed using G*Power with 200 subjects, an effect size, alpha error and power of 0.4, 0.05 and 0.99, respectively. Two orthopaedic surgeons measured the radiographs. The intraclass correlation coefficient (ICC) was used to assess interobserver reliability. We calculated 95% CI by using each value's mean and SE.

## RESULTS

**Figure 2:**
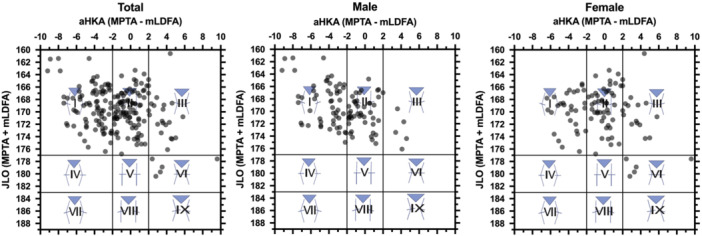
Distribution of CPAK types in the main analysis. aHKA, arithmetic hip knee angle; CPAK, coronal plane alignment of knee; JLO, joint line obliquity.

The ICC for each value was 0.94, 0.83 and 0.65 in mHKA, mLDFA and MPTA, respectively. In the main outcome of this study, a weak positive correlation was found between mHKA and MPTS, but not with aHKA for all. A moderate positive correlation was found between MPTS and LPTS. Comparing each value between sexes, males had significantly lesser mHKA than females (−2.8° ± 2.3° vs. −1.0° ± 2.1°). mLDFA was significantly greater in males (85.9° ± 1.7°), and MPTA was significantly smaller (83.6° ± 2.6). The percentage of constitutional varus was significantly greater in males than in females (43.8% vs. 15.8%). MPTS was not significantly different between sexes, whereas LPTS was significantly greater in males (8.6° ± 2.9). See Tables 1 and 2 for detailed values for each parameter. CPAK types I (51.4%) and Ⅱ (49.5%) were the most common in males and females, respectively (Figure 2).

**Table 1:** Measurement results of male and female.

	All (*n* = 200)	Male (*n* = 105)	Female (*n* = 95)	*p* Value
mHKA (°)	−2.0 ± 2.4	−2.8 ± 2.3^a^	−1.0 ± 2.1^a^	<0.01
aHKA (°)	−1.4 ± 3.2	−2.3 ± 3.0	−0.4 ± 3.0	<0.01
Constitutional varus (％)	30.5	43.8^b^	15.8^b^	<0.01
mLDFA (°)	85.5 ± 2.0	85.9 ± 1.7^c^	85.2 ± 2.2^c^	0.01
MPTA (°)	84.2 ± 2.7	83.6 ± 2.6^d^	84.8 ± 2.6^d^	<0.01
MPTS (°)	9.7 ± 3.3	10.9 ± 3.7	9.4 ± 2.8	0.21
LPTS (°)	8.1 ± 3.0	8.6 ± 2.9^e^	7.6 ± 3.0^e^	0.01

*Note*: mHKA and aHKA were defined as positive and negative values for valgus and varus, respectively.

Abbreviations: aHKA, arithmetic hip knee angle; LPTS, lateral posterior tibial slope; mHKA, mechanical hip knee angle; mLDFA, mechanical lateral distal femoral angle; MPTA, medial proximal tibial angle; MPTS, medial posterior tibial slope.

^a^ mHKA was significantly different between the sexes.

^b^ Constitutional varus proportion was significantly different between the sexes.

^c^ mLDFA was significantly different between the sexes.

^d^ MPTA was significantly different between the sexes.

^e^ LPTS was significantly different between the sexes.

**Table 2:** The correlation coefficient between each value.

	Correlation coefficient	*p* Value
mHKA–aHKA	−0.631^a^	<0.01
mHKA–mLDFA	0.31^b^	<0.01
mHKA– MPTA	−0.52^c^	<0.01
mHKA–MPTS	0.21^d^	<0.01
mHKA–LPTS	0.04	0.61
mLDFA–aHKA	−0.54^e^	<0.01
mLDFA–MPTA	0.10	0.14
mLDFA–MPTS	0.16	0.02
mLDFA–LPTS	0.05	0.51
MPTA–aHKA	0.79^f^	<0.01
MPTA–MPTS	−0.10	0.14
MPTA–LPTS	0.01	0.87
MPTS–aHKA	−0.19	<0.01
MPTS–LPTS	0.43^g^	<0.01
LPTS–aHKA	−0.02	0.79

Abbreviations: aHKA, arithmetic hip knee angle; LPTS, lateral posterior tibial slope; mHKA, mechanical hip knee angle; mLDFA, mechanical lateral distal femoral angle; MPTA, medial proximal tibial angle; MPTS, medial posterior tibial slope.

^a^ A moderate negative correlation was found between mHKA and aHKA.

^b^ A weak positive correlation was found between mHKA and mLDFA.

^c^ A moderate negative correlation was found between mHKA and MPTA.

^d^ A weak positive correlation was found between mHKA and MPTS.

^e^ A moderate negative correlation was found between mLDFA and aHKA.

^f^ A strong positive correlation was found between MPTA and aHKA.

^g^ A moderate positive correlation was found between MPTS and LPTS.

**Figure 3:**
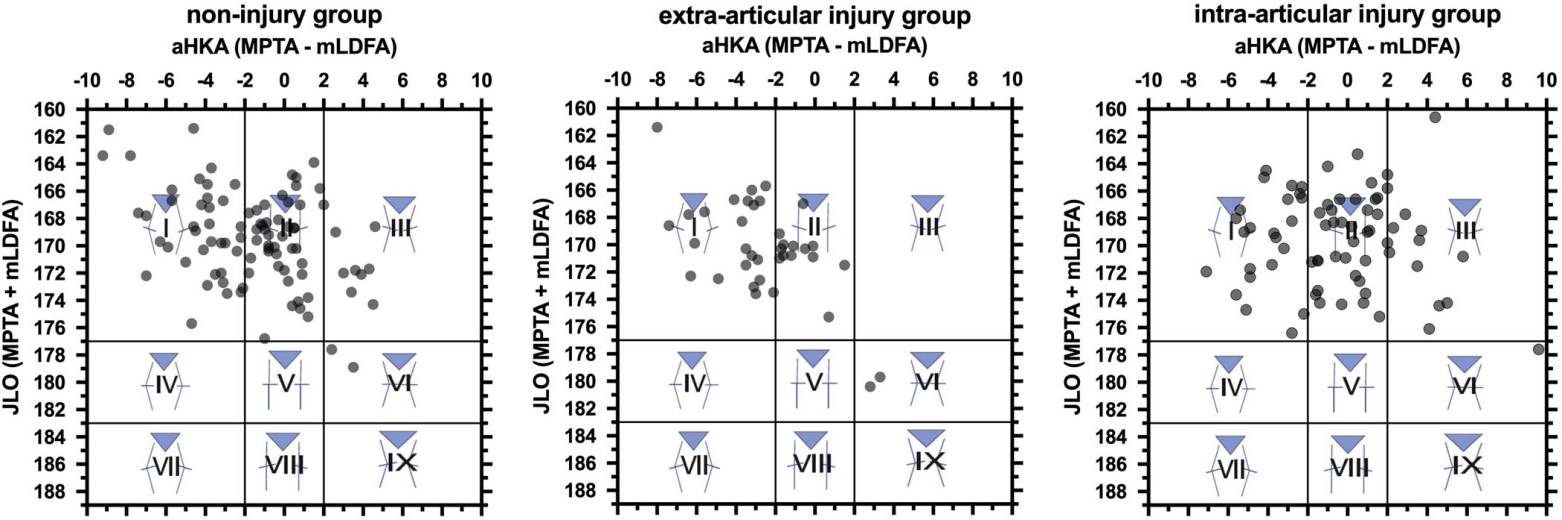
Distribution of CPAK types in the subgroup analysis. aHKA, arithmetic hip knee angle; CPAK, coronal plane alignment of knee; JLO, joint line obliquity.

In the secondary outcome, the subjects were followed up and divided into three subgroups (noninjury, intra‐articular injury and extra‐articular injury groups) based on the presence and location of injury. The extra‐articular group had significantly lesser aHKA than the intra‐articular group. The extra‐articular group had significantly greater mLDFA (86.5° ± 1.4°) than the other two groups. See Table 3 for detailed values for each parameter. The CPAK type frequency significantly differed between the extra‐ and intra‐articular groups. The extra‐articular group had a higher proportion of CPAK type I than the intra‐articular group (60.5% vs. 31.9%), but CPAK type Ⅱ was higher in the intra‐articular group (34.2% vs. 51.4%) (Figure 3).

**Table 3:** Measurement results of the subgroup analysis.

	Noninjury (*n* = 92)	Extra‐articular injury (*n* = 38)	Intra‐articular injury (*n* = 72)	*p* Value
mHKA (°)	−1.9 ± 2.2	−2.7 ± 2.6	−1.7 ± 2.4	0.09
aHKA (°)	−1.5 ± 3.1	−2.8 ± 2.8^a^	−0.5 ± 3.2^a^	<0.01
Constitutional varus (％)	29.3	44.7	23.6	0.12
mLDFA (°)	85.5 ± 1.8^b^	86.5 ± 1.4^b^, ^c^	85.1 ± 2.2^b^, ^c^	<0.01
MPTA (°)	84.0 ± 2.7^c^	83.7 ± 2.9	84.6 ± 2.5	0.21
MPTS (°)	9.5 ± 3.2	10.3 ± 3.6	9.6 ± 3.3	0.42
LPTS (°)	8.1 ± 3.1	8.6 ± 2.8	7.9 ± 2.9	0.50

Abbreviations: aHKA, arithmetic hip knee angle; LPTS, lateral posterior tibial slope; mHKA, mechanical hip knee angle; mLDFA, mechanical lateral distal femoral angle; MPTA, medial proximal tibial angle; MPTS, medial posterior tibial slope.

^a^ Significant difference between aHKA of the extra‐articular and intra‐articular injury groups (*p *< 0.01).

^b^ Significant difference between mLDFA of the noninjury and extra‐articular injury groups (*p *< 0.01).

^c^ Significant difference between mLDFA of the extra‐articular and intra‐articular injury groups (*p *< 0.01).

## DISCUSSION

The study examined the correlation between coronal and sagittal parameters as the primary endpoint, based on the hypothesis that there is little correlation between coronal and sagittal alignment in patients with knee complaints but without KOA. Our main findings show that in patients with knee complaints but without KOA, a weak positive correlation was found between mHKA and MPTS, but not with aHKA.

First, we examined the data obtained in this study for coronal and sagittal parameters and the trends of previous studies. Previous studies have shown that mHKA, mLDFA and MPTA differ by race, with Asians having greater mHKA and tibial varus than Europeans and Americans [8, 22]. PTS tended to be greater in males than in females, with greater MPTS in Asians than in Caucasians and varying values for LPTS [2, 3, 5, 17]. In the CPAK classification, males tended to have more varus alignment (CPAK type I) and females had more neutral alignment (CPAK types Ⅱ and V) than males, consistent with previous studies. The trends in coronal and sagittal parameters obtained in this study did not differ significantly from these trends. A comparison between this study and a previous study of healthy Japanese volunteers with 95% CI showed significant differences in mHKA, mLDFA and MPTA in females and mLDFA in males. The reason for the significant differences could be that we have not examined the aHKA. Few studies have examined MPTS and LPTS in the Japanese population, and comparisons using 95% CI were impossible.

Several previous studies examining the relationship between coronal and sagittal alignment have reported that there is little correlation between coronal and sagittal alignment in patients with KOA. Meier et al. reported a weak positive correlation between MPTA and MPTS in European patients scheduled for knee joint replacement surgery but with high interindividual variability [15]. Corbett et al. reported that there is little correlation between sagittal and rotational alignment and CPAK types in patients with KOA [4]. According to Ziegenhorn et al., there was a correlation between femoral torsion and coronal alignment in a group of patients before hip or knee replacements [23]. Our study may show that there is little correlation between coronal and sagittal alignment, even in a group of patients with knee complaints but without KOA.

In subgroup analysis, we divided the analysis into three groups: noninjury, intra‐articular injury and extra‐articular injury groups. The extra‐articular injury group had significantly lesser distal femoral valgus than the noninjury and intra‐articular injury groups. In addition, they had a higher proportion of CPAK type I than the intra‐articular injury group. Runner's knee was the most common in the extra‐articular injury group in this study; genu varum is a known risk factor for runner's knee and may be associated [11]. Further analysis of these separately in future studies may clarify lower limb alignment characteristics for each injury.

This study has some limitations. First, we subjected only patients with knee complaints and did not compare them with healthy volunteers in our institution, which could have more accurately analysed the lower limb alignment characteristics of the group of patients with knee complaints. Second, we only used radiographs as an assessment method. Three‐dimensional deformities, such as lower limb rotation and bowing, may affect the measurement results, and radiograph‐only assessment may be less accurate than computed tomography or magnetic resonance imaging. The joint line convergence angle, which may affect mHKA values, has not been measured and examined. We calculated and evaluated aHKA from mLDFA and MPTA. Third, measuring PTS can be measured in many ways, and previous studies have used different measurement methods. Furthermore, it is possible that there could be measurement errors in the MPTS and LPTS. In this study, only those with clearly distinguishable medial and lateral joint line surfaces in the lateral view were included.

## CONCLUSIONS

Little correlation was found between the coronal and sagittal parameters in patients with knee complaints but without KOA.

## AUTHOR CONTRIBUTIONS

The conception and design of this study were performed by Mikiko Handa, Tsuneari Takahashi and Masaki Iguchi. The acquisition of data was done by Mikiko Handa and Tsuneari Takahashi. Analysis and/or interpretation of data was carried out by Mikiko Handa, Tsuneari Takahashi and Akihiro Saitsu. The drafting of the article was done by Mikiko Handa, Tsuneari Takahashi and Katsushi Takeshita. All authors have contributed significantly to the study, approved the article and agreed with the submission.

## CONFLICT OF INTEREST STATEMENT

The authors declare no conflicts of interest.

## ETHICS STATEMENT

This study was performed in accordance with the Declaration of Helsinki and was approved by the Jichi Medical University Bioethics Committee for Medical Research (Approval ID: 22‐199). This study was retrospective. All patients received standard treatment, and the requirement for informed consent from individual participants was waived.

## Data Availability

Data and materials of this study are available from the corresponding author on reasonable request.
